# Five‐year survival post hepatectomy for colorectal liver metastases in a real‐world Chinese cohort: Recurrence patterns and prediction for potential cure

**DOI:** 10.1002/cam4.5732

**Published:** 2023-02-27

**Authors:** Yu‐Ming Su, Wei Liu, Xiao‐Luan Yan, Li‐Jun Wang, Ming Liu, Hong‐Wei Wang, Ke‐Min Jin, Quan Bao, Kun Wang, Juan Li, Da Xu, Bao‐Cai Xing

**Affiliations:** ^1^ Key Laboratory of Carcinogenesis and Translational Research (Ministry of Education/Beijing), Hepatopancreatobiliary Surgery Department I Peking University Cancer Hospital & Institute Beijing China

**Keywords:** colorectal cancer, hepatectomy, liver metastasis, survival

## Abstract

**Background:**

Patients with a 5‐year recurrence‐free survival post liver resection for colorectal cancer liver metastases (CRLM) are considered to be potentially cured. However, there is a deficit of data on long‐term follow‐up and the recurrence status among these patients in the Chinese population. We analyzed real‐world follow‐up data of patients with CRLM who underwent hepatectomy, explored the recurrence patterns, and established a prediction model for a potential cure scenario.

**Methods:**

Patients who underwent radical hepatic resection for CRLM during 2000–2016, with actual follow‐up data for at least 5 years, were enrolled. The observed survival rate was calculated and compared among the groups with different recurrence patterns. The predictive factors for 5‐year non‐recurrence were determined using logistic regression analysis; a recurrence‐free survival model was developed to predict long‐term survival.

**Results:**

A total of 433 patients were included, of whom 113 patients were found non‐recurrence after 5 years follow‐up, with a potential cure rate of 26.1%. Patients with late recurrence (>5 months) and lung relapse showed significantly superior survival. Repeated localized treatment significantly improved the long‐term survival of patients with intrahepatic or extrahepatic recurrences. Multivariate analysis showed that RAS wild‐type CRC, preoperative CEA <10 ng/ml, and liver metastases ≤3 were independent factors for a 5‐year disease‐free recurrence. A cure model was developed based on the above factors, achieving good performance in predicting long‐term survival.

**Conclusions:**

About one quarter patients with CRLM could achieve potential cure with non‐recurrence at 5‐year after surgery. The recurrence‐free cure model could well distinguish the long‐term survival, which would aid clinicians in determining the treatment strategy.

## Graphical abstract

With the advances in treatment for colorectal liver metastases (CRLM), accurately predicting recurrence‐free survival post hepatectomy has become increasingly important. This study retrospectively analyzed patients with CRLM who underwent surgery with actual follow‐up over 5 years; we found that approximately one quarter of patients got a potential cure chance without recurrence. Patients with RAS wild‐type, preoperative CEA <10 ng/mL, and liver metastases ≤3 were shown to have a higher chance of cure rate after hepatic resection for CRLM.

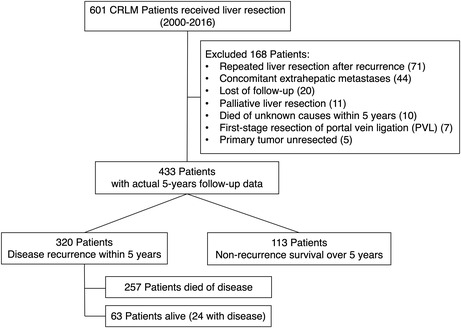

## 1 INTRODUCTION

The incidence of colorectal cancer (CRC) is increasing annually; it is the second most common cancer and has the fifth highest mortality rate in China.^1^ More than 50% of patients are estimated to develop liver metastases during the course of colorectal cancer.^2^, ^3^ Surgical resection is a curative treatment for long‐term survival in patients with colorectal liver metastases (CRLM), with a 5‐year survival rate of 40%–50%.^4^, ^5^, ^6^ With the improvement of surgical techniques and localized treatment in the last two decades, patients with larger tumor burdens were able to achieve long‐term survival through aggressive hepatectomy. Moreover, advancements in systemic chemotherapy and hepatic artery infusion have enabled more patients with initially unresectable liver metastases to qualify for tumor resection hepatectomy.^7^, ^8^, ^9^ However, with the expansion of hepatic resection criteria, more than 70% of patients develop recurrence postsurgery and their prognosis is significantly worse.^10^ Therefore, further exploration of recurrence patterns post hepatectomy and identification of populations at risk for recurrence are critical for effective treatment.

Most CRLM recurrences occur within 2 years after hepatectomy.^11^ The recurrence rate has gradually decreased with time. The probability of recurrence over 5 years postsurgery is quite low; therefore, a previous study considered nonrecurrence at 5 years postresection as cure.^12^ Approximately, 20% of patients achieved 5‐year recurrence‐free survival postliver resection, and the prognosis of these patients is excellent.^13^ Some studies used 10‐year overall survival as a surrogate for a curative scenario, considering that some patients would experience recurrence after 5 years.^14^ Besides, different factors including time of recurrence, site of recurrence, and treatment options after recurrence, have an impact on long‐term overall survival. Several studies have explored prognostic factors and developed scoring systems to predict recurrence or survival in patients with CRLM, which is helpful in stratifying prognosis.^15^, ^16^, ^17^ However, most studies included patients treated before 2000, which does not reflect patient characteristics and treatment in the modern era. In addition, some studies used predictive survival as an endpoint rather than long‐term follow‐up data.^18^, ^19^ Moreover, patients with concurrent extrahepatic metastases or non‐radical resection were included in previous studies, which could influence recurrence after hepatectomy. Therefore, previous models are insufficient to accurately reflect the actual cure rate and prognostic factors in Chinese patients with CRLM who underwent liver resection in the current situation. The purpose of this study was to analyze patients with limited‐liver CRLM who had been followed up for more than 5 years, explore the recurrence patterns, and develop a potential cure prediction model for patients with CRLM who underwent surgery in the modern era.

## 2 MATERIALS AND METHODS

### 2.1 Patient eligibility

Patients with limited‐liver CRLM who underwent radical hepatectomy between January 2000 and December 2016 with actual 5 years follow‐up data from our Hepatopancreatobiliary Surgery Department I at Beijing Cancer Hospital (Beijing, China) were retrospectively analyzed. The inclusion criteria were as follows: (1) A pathologically confirmed diagnosis of CRLM; (2) Underwent radical liver metastases surgery (including only surgical resection or surgical resection in combination with other localized treatments); (3) Patients with actual follow‐up for more than 5 years. Patients were excluded per the following criteria: (1) non‐radical hepatic resection or unresected colorectal tumor; (2) concomitant extrahepatic metastases; (3) missing clinical information or being lost to follow‐up; (4) repeated liver resection after recurrence; and (5) death due to unknown causes within 5 years. The requirement for informed consent was waived due to the retrospective design of the study. All the patients enrolled in this study were Chinese citizens. The clinicopathologic information was retrospectively collected from the center's CRLM surgery database. The primary study endpoints were long‐term survival outcomes and cure model establishment.

### 2.2 Treatment

Patients with CRLM routinely underwent gene test including KRAS, NRAS (exon 2, codons 12,13), and BRAF V600E status. Less than 10 patients were with BRAF mutation or MSI‐H in our research. To avoid the bias, we did not take these into analysis. Contrast‐enhanced MRI and chest and pelvic computed tomography (CT) scans were routinely performed before surgery. Positron emission tomography‐CT was performed in case extrahepatic disease was suspected. Liver metastases were considered resectable as long as complete ablation of liver metastases using surgical resection with R0/R1 margin or combined radiofrequency ablation, with >30% resultant liver remnants, >0.7 remnant liver‐to‐body weight ratio, and complete blood inflow and outflow could be maintained postsurgery. Neoadjuvant chemotherapy is recommended for patients with a high clinical risk score (CRS). Patients with CRS <3 were defined as low‐risk group, and patients with CRS≥3 were defined as high‐risk group. In case of a heavier tumor burden or anticipated difficulty in resection, conversion chemotherapy is administered to achieve resectability. Chemotherapy regimens included oxaliplatin‐ or irinotecan‐based chemotherapy (mFOLFOX6/CAPEOX/FOLFIRI/FOLFOXIRI), with or without targeted agents (bevacizumab and cetuximab). The time interval between the last chemotherapy session and hepatic surgery was usually 2–4 weeks, extending to 6–8 weeks with the addition of bevacizumab. When the liver metastases were technically easily resected or combined with small lesions that may disappear postchemotherapy, surgical resection may be directly performed. Primary tumors located in the cecum, ascending colon, and transverse colon were defined as right‐side (RS) tumors, and those located in the splenic flexure, descending colon, sigmoid colon, and rectum were defined as left‐side (LS) tumors. Imaging tools were used to confirm recurrence, including abdominal and pelvic contrast‐enhanced CT, chest CT, and liver enhanced magnetic resonance. Routinely, the examination is performed every 3 months within the 2 years after the liver resection, every 6 months at the 3–5 year and every 1 year over 5 years. At the same time, telephone follow‐up was performed twice every year. Recurrence time is recorded at the time of the recurrence reported by imaging tools. The definition of different recurrent organs is as below: liver recurrence is the recurrence limited to the liver; lung recurrence is the recurrence which first recur at the lung; bone, brain, or more than two organs is that whose recurrence at the bone, at the brain, at these two organs or at more than two organs; others are referred to that whose recurrence did not belong to any of the above. For patients with recurrence, treatments like resection, local treatments, or palliative chemotherapy were selected according to the recurrence patterns.

### 2.3 Statistical methods

The univariate comparisons of clinicopathologic variables were performed using the chi‐squared test, *t*‐test and Mann–Whitney U test. Recurrence‐free survival (RFS) was measured from the time of resection of liver metastases until recurrence or the last follow‐up. Overall survival (OS) was defined from the time of resection of liver metastases until death or the last follow‐up. Survival curves were plotted using the Kaplan–Meier method and were compared by the log‐rank test. A multivariate logistic regression analysis was used to identify the predictors of 5‐year nonrecurrence including variables that were statistically significant in the univariate analysis (*p* < 0.05). Receiver operating characteristic (ROC) curve was used to evaluate the diagnostic performance for predicting 5‐year recurrence; the area under the ROC curve (AUC) with its 95% confidence intervals (CI) was calculated. All statistical analyses were conducted using SPSS (version 26) and GraphPad Prism (Version 9.2.0). Statistical significance was set at *p* < 0.05.

## 3 RESULTS

### 3.1 Characteristics of patients

**FIGURE 1:**
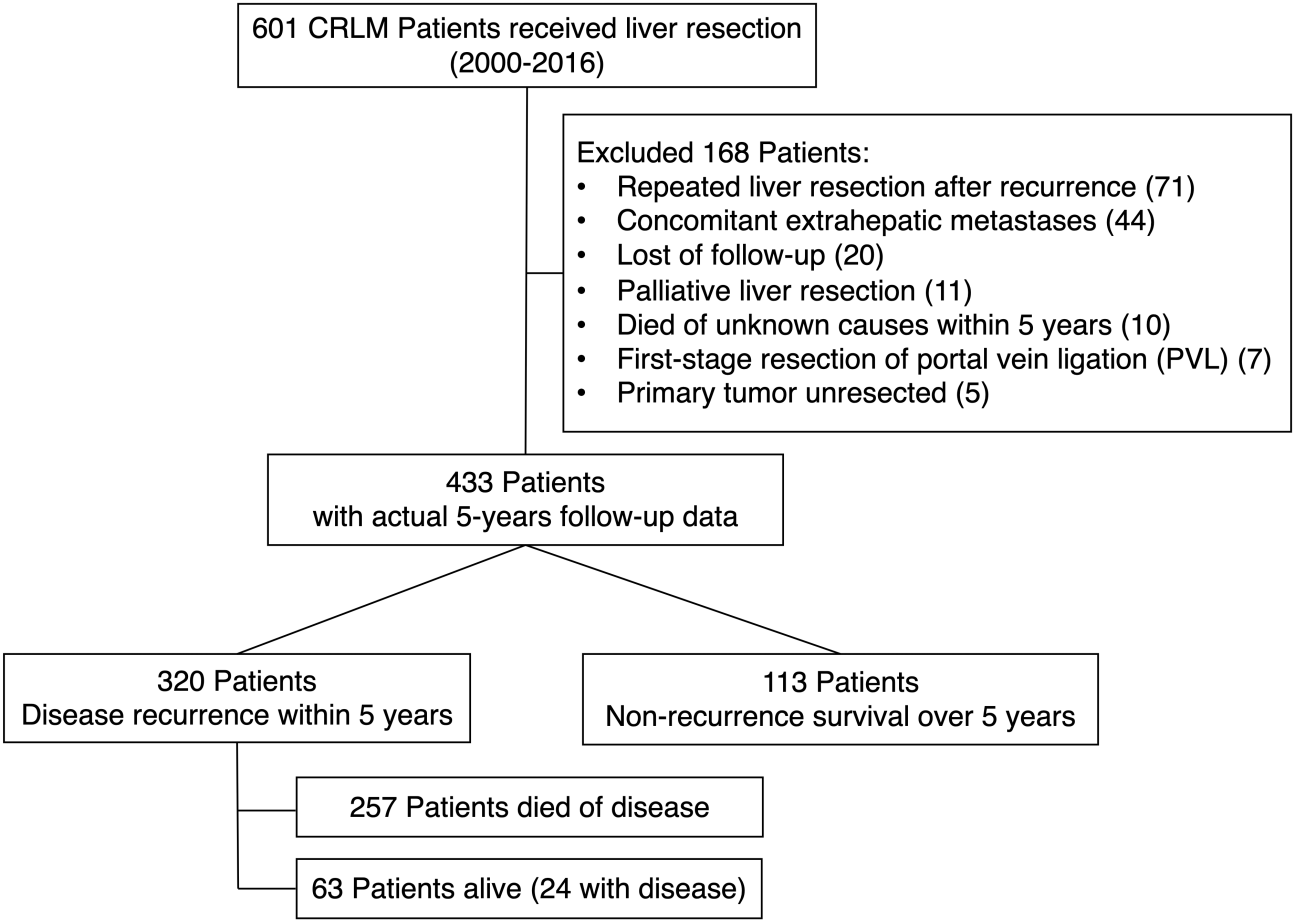
Flow diagram of patients with colorectal liver metastases who underwent liver resection.

A total of 601 patients with CRLM underwent hepatic resection from January 2000 to December 2016 at the Hepatopancreatobiliary Surgery Department I in the Beijing Cancer Hospital. One hundred and sixty‐eight patients were excluded per our criteria, including those who underwent repeated liver resection postrecurrence (*n* = 71), patients with concomitant extrahepatic metastases (*n* = 44), and patients who were lost to follow‐up (*n* = 20), patients who underwent palliative liver resection (*n* = 11), patients who died of unknown causes within 5 years (*n* = 10), patients who underwent first‐stage resection of portal vein ligation (PVL) (*n* = 7), and patients with unresected primary tumor (*n* = 5). Finally, a total of 433 patients with actual 5 years of follow‐up data were enrolled in this study. Among them, 320 patients had tumor recurrence within 5 years, in which 63 patients were alive (24 with disease). One hundred and thirteen patients had no recurrence after 5 years of follow‐up, with a potential cure rate of 26.1%. The flow diagram of the study is shown in Figure 1. The patients who experienced recurrence within 5 years had a higher proportion of *N* positive, synchronous liver metastases, >3 liver metastases, high‐risk CRS, RAS mutation, bilobar liver distribution, and higher preoperative CEA (Table 1).

**TABLE 1:** Univariate analysis of factors influencing 5 years nonrecurrence.

Variables	Recur within 5 years	Nonrecurrence within 5 years	OR	95% CI	*p*‐value
Age (mean, year)	56.61 ± 10.376	57.18 ± 10.862			0.624
Sex			0.709	0.449–1.121	0.141
Male	196 (61.25%)	78 (69.0%)			
Female	124 (38.75%)	35 (31.0%)			
Primary CRC T stage			1.946	0.998–3.797	0.051
T1–2	25 (7.8%)	16 (14.2%)			
T3–4	295 (92.2%)	97 (85.8%)			
Primary CRC *N* stage			**1.654**	**1.071–2.553**	**0.023**
*N* negative	114 (35.6%)	54 (47.8%)			
*N* positive	206 (64.4%)	59 (52.2%)			
Primary CRC location			0.788	0.444–1.401	0.418
Right side	62 (19.4%)	18 (15.9%)			
Left side	258 (80.6%)	95 (84.1%)			
Timing of liver metastasis			**1.880**	**1.218–2.901**	**0.004**
Synchronous	186 (58.1%)	48 (42.5%)			
Metachronous	134 (41.9%)	65 (57.5%)			
Liver metastasis size (mm)			1.516	0.835–2.749	0.171
<50	256 (80%)	97 (85.8%)			
≥50	64 (20%)	16 (14.2%)			
Liver metastasis number			**2.216**	**1.282–3.830**	**0.004**
≤3	215 (67.2%)	94 (83.2%)			
>3	105 (32.8%)	19 (16.8%)			
Distribution of liver metastases			**1.581**	**1.012–2.470**	**0.044**
Unilobar	174 (54.5%)	74 (65.5%)			
Bilobar	145 (45.5%)	39 (34.5%)			
RAS status			**1.839**	**1.115–3.032**	**0.017**
Wild type	190 (62.3%)	79 (75.2%)			
Mutastion^5^	115 (37.7%)	26 (24.8%)			
Preoperative CEA (ng/mL)			**1.763**	**1.127–2.759**	**0.013**
CEA <10	169 (52.8%)	75 (66.4%)			
CEA ≥10	151 (47.2%)	38 (33.6%)			
Preoperative CA19–9 (IU/mL)			1.544	0.912–2.615	0.104
CA19–9 < 50	233 (72.8%)	91 (80.5%)			
CA19–9 ≥ 50	87 (27.2%)	22 (19.5%)			
CRS			**2.348**	**1.476–3.736**	**<0.001**
Low risk (<3)	166 (51.9%)	81 (71.7%)			
High risk (≥3)	154 (48.1%)	32 (28.3%)			
Preoperative chemotherapy			1.403	0.906–2.171	0.128
Yes	207 (64.7%)	64 (56.6%)			
No	113 (35.3%)	49 (43.4%)			
Postoperative chemotherapy			0.566	0.325–0.984	0.044
No	85 (30.9%)	20 (20.2%)			
Yes	190 (69.1%)	79 (79.8%)			
Time period			**1.820**	**1.024–3.263**	**0.039**
Before 2010	78 (24.4%)	17 (15.0%)			
After 2010	242 (75.6%)	96 (85.0%)			

Abbreviations: CA19‐9, carbohydrate antigen 19–9; CEA, carcinoembryonic antigen; CRC, colorectal cancer; CRS, clinical risk scores. Bold values indicate statistically significant differences.

### 3.2 Actual long‐term survival

**FIGURE 2:**
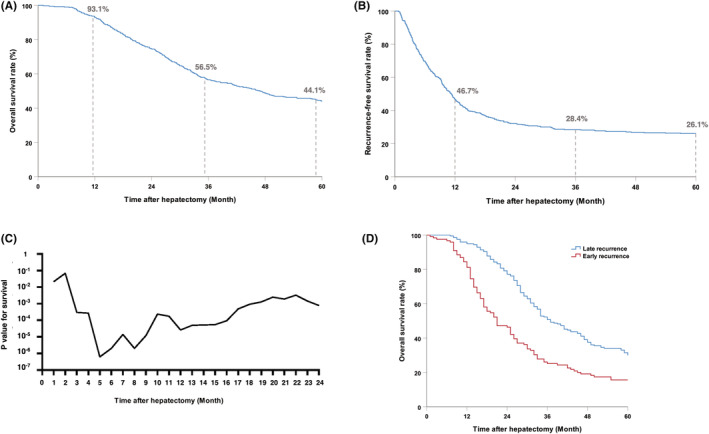
(A) Overall survival and (B) recurrence‐free survival of patients with actual 5‐years follow‐up post hepatectomy. When relapse occurred within 5 years, the impact of time of recurrence on overall survival was plotted using the log‐rank test (C) Recurrence within 5 months showed greatest impact in distinguishing the prognosis and was defined as the best cutoff for early recurrence (*p* = 6.13*10^−7^) (D).

The median follow‐up duration for the entire cohort was 80 months. The actual 1‐, 3‐, and 5‐year OS and RFS were 93.1%, 56.5%, 44.1%, and 46.7%, 28.4%, and 26.1%, respectively (Figure 2A,B). Among the patients who experienced recurrence within 5 years (73.9%), the proportion of recurrence in the 1st, 2nd, 3rd, 4th, and 5th years was 54.5%, 13.5%, 3.2%, 1.6%, and 0.7%, respectively (Figure S1). Patients who relapsed within the first 2 years accounted for 92% of the recurrence sample. Further analysis showed that relapse within 5 months after hepatectomy had the best performance in distinguishing the prognosis (*p* = 6.13*10^−7^) (Figure 2C). When definite early recurrence using a 5‐month cutoff value, patients were divided into early recurrence group and late recurrence group. The general clinical data of the two groups of patients are compared in Table S1. The overall survival of patients with early recurrence (≤5 months) was significantly worse than that of patients with late recurrence (>5 months) (5‐year OS rates 31.5% vs. 15.6%, *p* < 0.001) (Figure 2D).

### 3.3 Recurrence sites and subsequent treatment options

**FIGURE 3:**
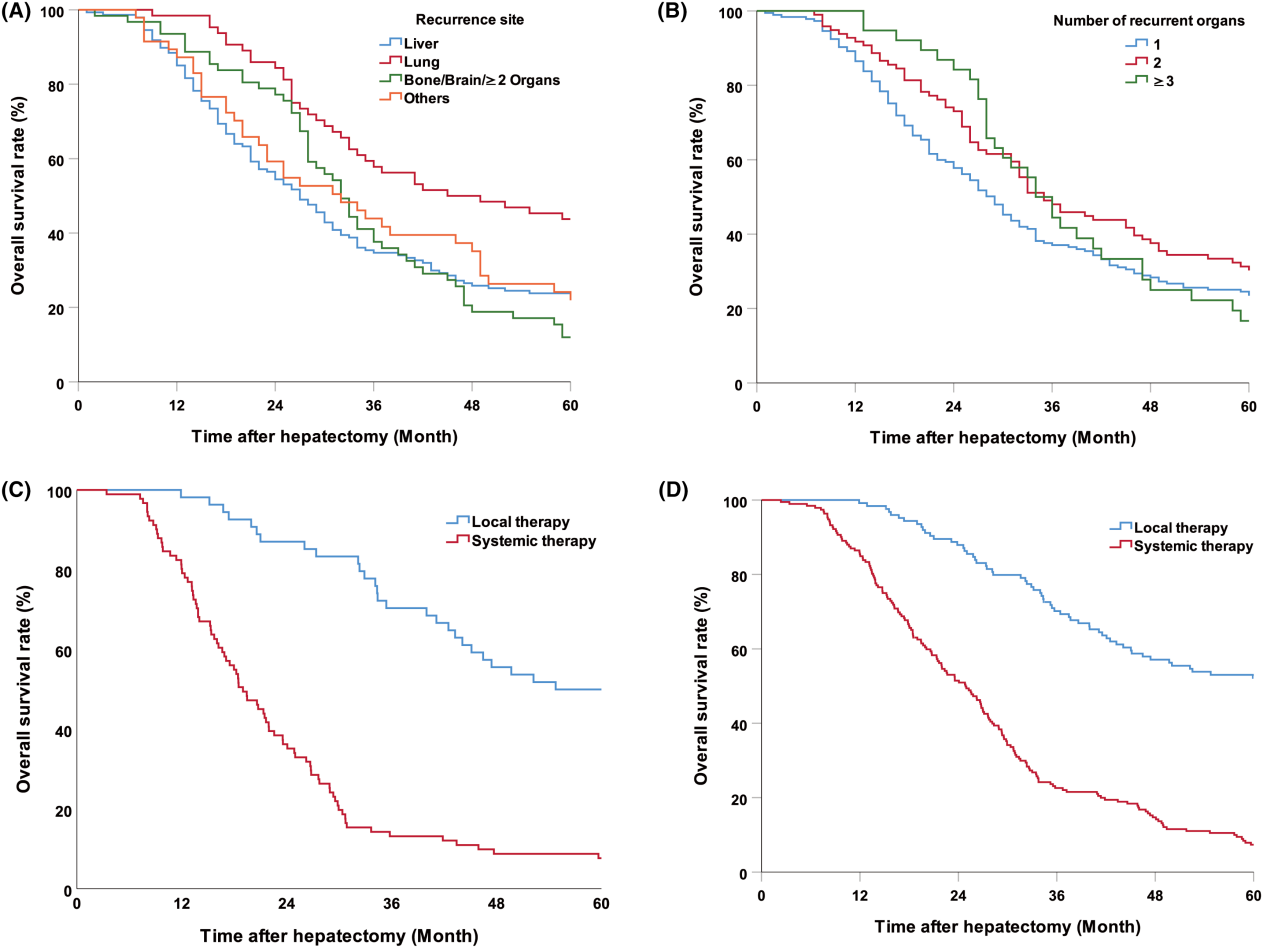
Overall survival of patients with (A) different first recurrence sites (lung vs liver, *p* < 0.001; lung vs. bone/brain, *p* < 0.001; lung versus other sites, *p* = 0.010; liver vs. bone/brain, *p* = 0.535; liver vs. other sites, *p* = 0.682) and (B) different number of organs involved in recurrence (*p* = 0.346). The impact of treatment strategy after recurrence between local therapy and palliative systemic therapy was compared in patients with intrahepatic recurrence (*p* < 0.001) (C) and intra‐ or extrahepatic recurrence (*p* < 0.001) (D).

In patients who developed recurrence within 5 years, the most common site of first recurrence was the liver (147, 45.9%), followed by the lung (64, 20.0%), bone–brain or multiple organs (62, 19.4%), and other sites, including the peritoneum, abdominal lymph nodes, anastomoses, adrenals, and ovaries (47, 14.7%) (Figure S2). Patients with lung recurrence had the best prognosis compared to patients with recurrence in other sites (lung vs. liver, *p* < 0.001; lung vs bone/brain, *p* < 0.001; lung vs other sites, *p* = 0.010); there was no difference between the prognoses of patients with intrahepatic recurrences and that of patients with recurrences in other site (liver vs. bone/brain, *p* = 0.535; liver vs. other sites, *p* = 0.682) (Figure 3A). However, the number of organs involved in recurrence (1, 2, and 3 or more) did not have a significant impact on prognosis (*p* = 0.346) (Figure 3B). In order to further clarify the impact of postrecurrence treatments on the long‐term survival, we explored the prognosis of patients with intrahepatic or extrahepatic recurrence postsurgery, who received localized treatment or palliative therapy. The results showed that for patients with either intrahepatic or extrahepatic recurrence, repeated surgery, or localized treatment significantly improved long‐term survival compared to palliative chemotherapy (intrahepatic recurrence, *p* < 0.001; intra‐ and extrahepatic recurrence, *p* < 0.001) (Figure 3C,D).

### 3.4 Factors of postoperative nonrecurrence and development of a cure model

**FIGURE 4:**
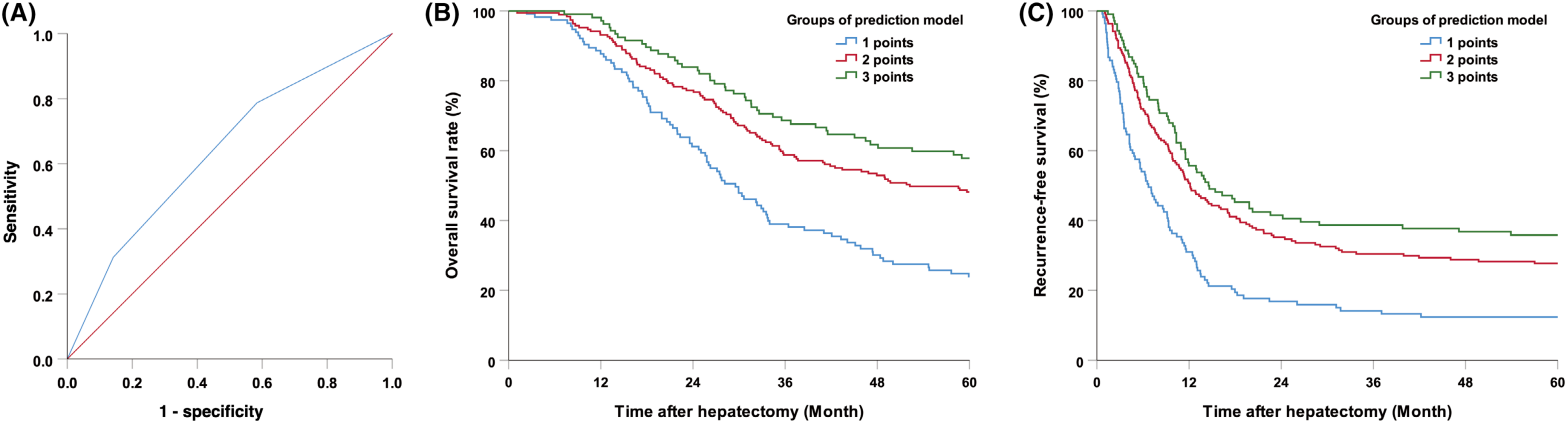
(A) Receiver operating characteristic (ROC) curve of the recurrence‐free cure model. Overall survival (B) and recurrence‐free survival (C) after hepatectomy for patients with different groups of prediction model (all *p* < 0.001).

In the univariate analysis, primary *N* negativity, presence of RAS wild‐type CRC, metachronous metastases, liver metastases ≤3, single lobe metastasis distribution, and CEA < 10 ng/mL were identified as predictors of nonrecurrence at 5 years (Table 1). Multivariate analysis further determined that presence of RAS wild‐type CRC (odds ratio (OR) (95% CI):1.900 [1.134–3.185], *p* = 0.015), liver metastases ≤3 (OR (95% CI):2.204 [1.138–4.267], *p* = 0.019), and preoperative CEA < 10 ng/mL (OR (95% CI):1.671, 95% CI 1.035–2.698, *p* = 0.036) were independent predictors for nonrecurrence at 5‐year (Table 2). A recurrence‐free cure model (RFS score) was established by combining the above three factors and assigning each factor one point. The ROC curve for the model predicting 5‐year nonrecurrence was determined. The area under the curve (AUC) was 0.637 (95% CI: 0.578–0.696) (Figure 4A). The 5‐year RFS and OS for patients with three points, any of the two points, and ≤ 1 point were 40.9%, 24.8%, and 13.8% (*p* < 0.001) and 59.4%, 46.8%, and 24.4% (*p* < 0.001), respectively (Figure 4B,C).

**TABLE 2:** Multivariate analysis to identify independent factors for 5 years nonrecurrence.

Variables	OR	95% CI	*p*
CRC *N* stage (N0 vs. N+)	1.502	0.939–1.502	0.090
Timing of liver metastases (metachronous vs. synchronous)	1.521	0.934–2.477	0.092
CRLM number (≤3 vs. >3)	**2.204**	**1.138–4.267**	**0.019**
CRLM Distribution (unilobar vs. bilobar)	1.001	0.586–1.709	0.997
RAS status (Wild type vs. mutation)	**1.900**	**1.134–3.185**	**0.015**
CEA (<10 vs ≥ 10 ng/mL)	**1.671**	**1.035–2.698**	**0.036**

Abbreviations: CEA, carcinoembryonic antigen; CRC, colorectal cancer; CRLM, colorectal cancer liver metastasis. Bold values indicate statistically significant differences.

## 4 DISCUSSION

With the development of effective systemic therapy and aggressive localized surgical treatment in recent years, more patients with advanced CRLM achieved long‐term survival, which is significantly superior to palliative chemotherapy.^20^ This study analyzed the actual follow‐up data of patients with CRLM who underwent hepatic resection and found that approximately one quarter of the patients obtained a 5‐year recurrence‐free survival, which means a potential cure. By analyzing the predictors of 5‐year nonrecurrence, an RFS model was established, which was effective in predicting survival. Further analysis of the relapsed population showed that the time of recurrence, recurrence site, and treatment administered after relapse could also affect long‐term survival. These results will aid clinicians in developing better treatment strategies and in the selection of patients for surgical treatment.

Previous studies have explored predictors of long‐term survival in patients with CRLM after hepatectomy. However, these studies are based on predicted survival rather than the actual survival rate. A previous study in 2015 analyzed the data of 1012 consecutive patients who underwent curative resection for CRLM during 2001–2012.^19^ Another study published in 2018 enrolled patients undergoing consecutive liver resections for colorectal metastases between 1994 and 2014, although follow‐up <10 years were not included in this analysis.^21^ This might affect the results due to the censoring of patients and insufficient follow‐up time. Some studies also included patients treated before the year 2000, when systemic treatment and genetic testing were not widely used.^14^, ^22^ Therefore, accurate and relevant data on the factors affecting long‐term survival post hepatectomy in the current era of systemic chemotherapy. Moreover, about 7%–11% of patients included in the previous studies had concomitant extrahepatic disease, which were confirmed to be an independent factor affecting prognosis.^18^, ^22^ However, patients with extrahepatic metastases might have already developed minimal residual disease, which might also affect true long‐term survival cure rate and the determination of the prognostic factors postliver resection. In this study, all included patients were treated after 2000, when systemic therapy was widely used for CRLM treatment. Most patients underwent KRAS/NRAS/BRAF gene testing before treatment or were retrospectively tested. In addition, all included patients had metastasis limited to the liver and had at least 5 years of follow‐up survival data, which helped us to determine prognostic factors more accurately.

Due to the heterogeneity of tumor differentiation types, tumor stage, and treatment methods, there is currently no gold standard for the definition of a cure for cancer. Some studies have explored the 10‐year survival after liver resection to define a cure for patients with CRLM. Creasy JM et al. analyzed 1211 patients with a median follow‐up for 11 years, with the observed cure rate of 20.6% (*n* = 250), including 58 patients (23.2%) who had recurrence during follow‐up.^22^ Buisman FE et al. conducted a multicenter study including 4112 patients with an estimated 10‐year OS of 30% after resection of CRLM.^18^ These studies included patients with tumor relapse during follow‐up, after which they underwent treatment for the relapsed lesions to achieve long‐term survival. Tumor recurrence during follow‐up after radical resection usually indicates minimal residual metastases in the body, which may not be a true cure. In this study, the actual follow‐up of 5 years without recurrence was used as the endpoint, which might more accurately reflect the residual status of the patients. Although previous studies showed that a few patients would still have tumor relapse 5 years after surgery, only 0.4% of patients relapsed during the follow‐up after 5 years in this study, which is relatively low and has less impact on overall survival outcomes. A previous study also found that hazard ratios for tumor recurrence tends to decrease until it approaches zero at end of 6 years after surgery, which can be considered “cured” with 99% certainty.^19^ In fact, the 5‐year recurrence‐free rate (26.1%) in this study was close to the 10‐year overall survival rate (20–30%) reported in previous studies.^14^, ^18^, ^19^, ^21^, ^22^, ^23^ Moreover, 5 years follow‐up period was relatively short and easy to implement clinically. Thus, we believe that 5‐year nonrecurrence is a good indicator for evaluating the benefit of surgery for CRLM.

The time till recurrence after liver metastasis resection is considered an important factor affecting prognosis. Our study showed that most patients experienced recurrence within 5 years, especially within 2 years of surgery. This is also related to the biological characteristics of CRLM. Since these patients are stage IV colorectal cancer patients who already have metastatic disease, micrometastatic lesions may be present that cannot be detected by imaging, which might lead to early recurrence postsurgery. Previous study found that the tumor relapse within 8 months showed poorer biological behavior and conveyed significantly worse survival, which is a better cutoff to distinguish early and late recurrence.^10^ In this study, the time till recurrence with the greatest impact on overall survival was 5 months, which is determined by the smallest *p*‐value method. Patients who experienced recurrence within 5 months had significantly poorer survival. Sites of recurrence and treatment after recurrence are also important factors influencing survival. This study found that patients who first presented with lung relapse showed superior overall survival than those with relapse in the liver, bone/brain, or other multisite recurrences after radical surgery for CRLM. Several studies have suggested that prognosis varies among patients with different recurrence sites.^24^, ^25^, ^26^ Lung metastases often progresses slowly and exhibits more favorable biological behavior.^27^ In addition, active localized treatment could significantly prolong long‐term survival in patients with either intrahepatic or extrahepatic recurrences. The results were consistent with those of previous studies showing that repeated localized treatment for selected patients with recurrence could help achieve long‐term survival outcomes similar to those of the first surgery.^28^, ^29^, ^30^

Several prognostic scoring systems have been established to evaluate long‐term survival after surgery for CRLM. The CRS scoring system proposed by Fong et al. is a commonly used recurrence risk assessment system.^15^ However, it was developed based on patients treated before 2000 when chemotherapy and gene testing were not routinely performed. Many subsequent models, such as m‐CS, GAME, combined gene status, and tumor burden, have been used to comprehensively evaluate patient prognosis.^31^, ^32^, ^33^ Most of these models used overall survival time as the predictive endpoint. In fact, in addition to the clinical characteristics of the patient, many other factors, such as the recurrence pattern and treatment, would have an impact on long‐term survival. In this study, 5‐year recurrence‐free survival was used as a predictive endpoint, which could more simply and clearly identify the factors affecting recurrence and the biological behavior of the tumor. Among the predictive factors identified, tumor number and preoperative CEA levels were consistent with those reported in previous studies. In addition, the RAS status was found to significantly influence recurrence. Creasy et al. did not include this factor because the years and scope of this project did not allow for this correlation.^22^ Buisman et al. also found KRAS mutation status to be a prognostic factor for 10‐year OS; however, 61.9% of patients in the study had missing data, which might have affected the results.^18^ Previous studies also found that tumor size, primary tumor stage, and chemotherapy regimen influence recurrence.^14^, ^15^, ^17^, ^18^, ^21^ However, these factors were not independent predictors of 5‐year nonrecurrence in this study. The potential reasons for these discrepancies might be related to the differences in the baseline characteristics of the enrolled populations between studies and the differences in the enrolled ethnic groups. It should be emphasized that the recurrence‐free cure model could be used as a reference for survival, but it could not absolutely preclude 5‐year nonrecurrence survival, even in the high score group. It is helpful for clinicians to better judge the biological behavior of patients preoperatively and make clinical decisions regarding enthusiasm for treatment according to the RFS model.

The main limitation of this study is that it was a retrospective single‐center real‐world study with a limited sample size, which may have led to biases in the results. In addition, this study did not include patients with extrahepatic metastases at the time of liver resection and those who did not undergo radical resection; this was because even if patients with extrahepatic metastases underwent radical resection, they might have already developed extensive metastases, which makes it hard to determine whether it is postoperative recurrence or unresected minimal residual disease itself. Thus, we excluded these patients that might have an impact on the long‐term survival of true cure rate after hepatectomy and the factors that affect relapse. Commonly used laboratory examination results and clinicopathologic factors were all included in the analysis since they were important for prediction of prognosis and monitoring recurrence. The BRAF mutation status was also tested in combination with RAS at our center. However, since only two patients were found to have a mutation in the study cohort, it was not analyzed as a predictor. Finally, due to the long time span of the study, changes in chemotherapy regimens and local treatment technique advancements might also have impacted long‐term survival. For example, more patients received aggressive systemic chemotherapy regimens, such as targeted drugs or triplet chemotherapy, which might have increased the response rate and tumor regression rate. In addition, more extensive surgical strategy or surgery in combination with other localized treatments such as ablation or stereotactic radiotherapy, allows patients with heavier tumor burdens to have the opportunity to undergo surgery, which might also have an impact on long‐term survival. An expanded larger sample size with multicenter external validation is needed in the future.

In conclusion, despite the high rate of recurrence postsurgery among patients with CRLM, liver resection could significantly improve survival, achieving a potential cure of more than 20%. RFS score can distinguish the long‐term survival of patients. A total of 40% of patients with high scores could achieve 5‐year nonrecurrence, while only 10% with low scores were able to achieve the same results. For patients with tumor relapse, late recurrence (>5 months) and lung metastases were associated with better survival rates. Repeated localized treatment could improve long‐term prognosis in patients with intrahepatic or extrahepatic recurrence. Our results suggest that CRLM is a special type of advanced metastatic tumor with good prognosis. Clinicians and patients should strive to seek localized treatments and fully prepare for long‐term chronic antitumor treatment based on recurrence patterns.

## AUTHOR CONTRIBUTIONS

**Yu #x2010;Ming Su:** Conceptualization (equal); data curation (lead); formal analysis (lead); methodology (equal); resources (equal); software (equal); validation (equal); writing – original draft (equal); writing – review and editing (equal). **Wei Liu:** Data curation (equal); resources (equal). **Xiao #x2010;Luan Yan:** Data curation (equal). **Li #x2010;Jun Wang:** Data curation (equal); resources (equal). **Ming Liu:** Data curation (equal); resources (equal). **Hong #x2010;Wei Wang:** Data curation (equal); resources (equal). **Ke #x2010;Min Jin:** Data curation (equal); resources (equal). **Quan Bao:** Data curation (equal); resources (equal). **Kun Wang:** Data curation (equal); funding acquisition (equal); project administration (equal); resources (equal); supervision (equal). **Juan Li:** Data curation (equal); resources (equal). **Da Xu:** Conceptualization (equal); data curation (equal); formal analysis (equal); investigation (equal); methodology (equal); project administration (equal); resources (equal); software (equal); supervision (equal); validation (equal); visualization (equal); writing – original draft (equal); writing – review and editing (equal). **Bao #x2010;Cai Xing:** Conceptualization (equal); funding acquisition (equal); methodology (equal); project administration (equal); supervision (equal); writing – review and editing (equal).

## FUNDING INFORMATION

This study was funded by grants (No. 81874143, No. 31971192) from the National Nature Science Foundation of China, the Beijing Natural Science Foundation (No. 7192035), and the Beijing Capital's Funds for Health Improvement and Research (CFH, No.2022–1‐2151).

## CONFLICT OF INTEREST STATEMENT

All of the authors declare that they have no conflict of interests.

## ETHICS STATEMENT

The study protocol was approved by the Medical Ethics Committee of Beijing Cancer Hospital, and written informed consent was waived due to its retrospective collection of clinical information. Patients' privacies were well protected.

## Supporting information


Figure S1.

Figure S2.

Table S1.

## Data Availability

The original data of this study can be obtained on request from the corresponding author.
