# Supplementary figures and images for: Five‐year survival post hepatectomy for colorectal liver metastases in a real‐world Chinese cohort: Recurrence patterns and prediction for potential cure
Source: Cancer Med. 2023 Feb 27;12(8):9559–69. doi: 10.1002/cam4.5732 (PMC10166917; doi:10.1002/cam4.5732)

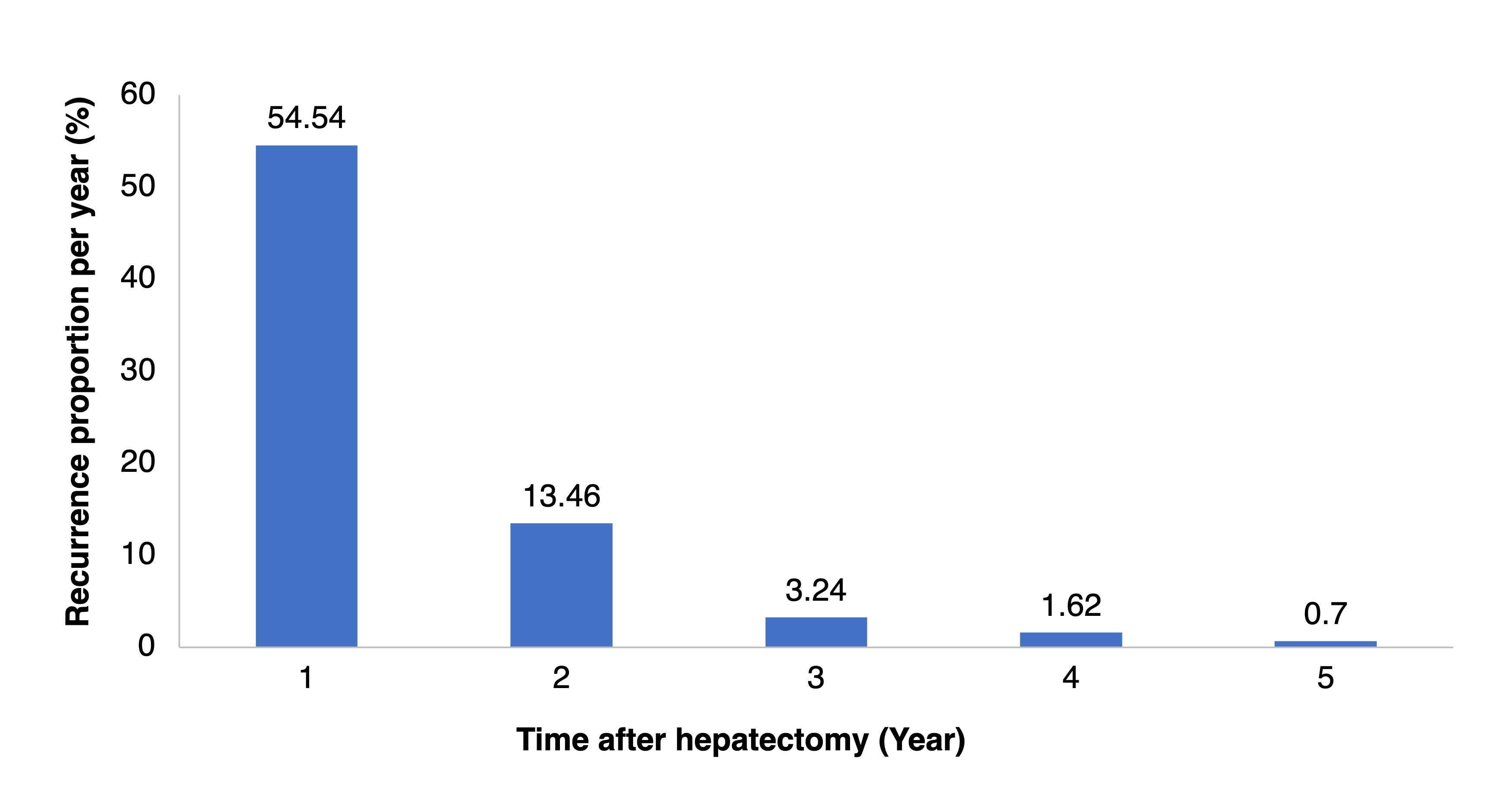

Supplement: Supplementary file 1 — Figure S1. [file CAM4-12-9559-s003.tiff]

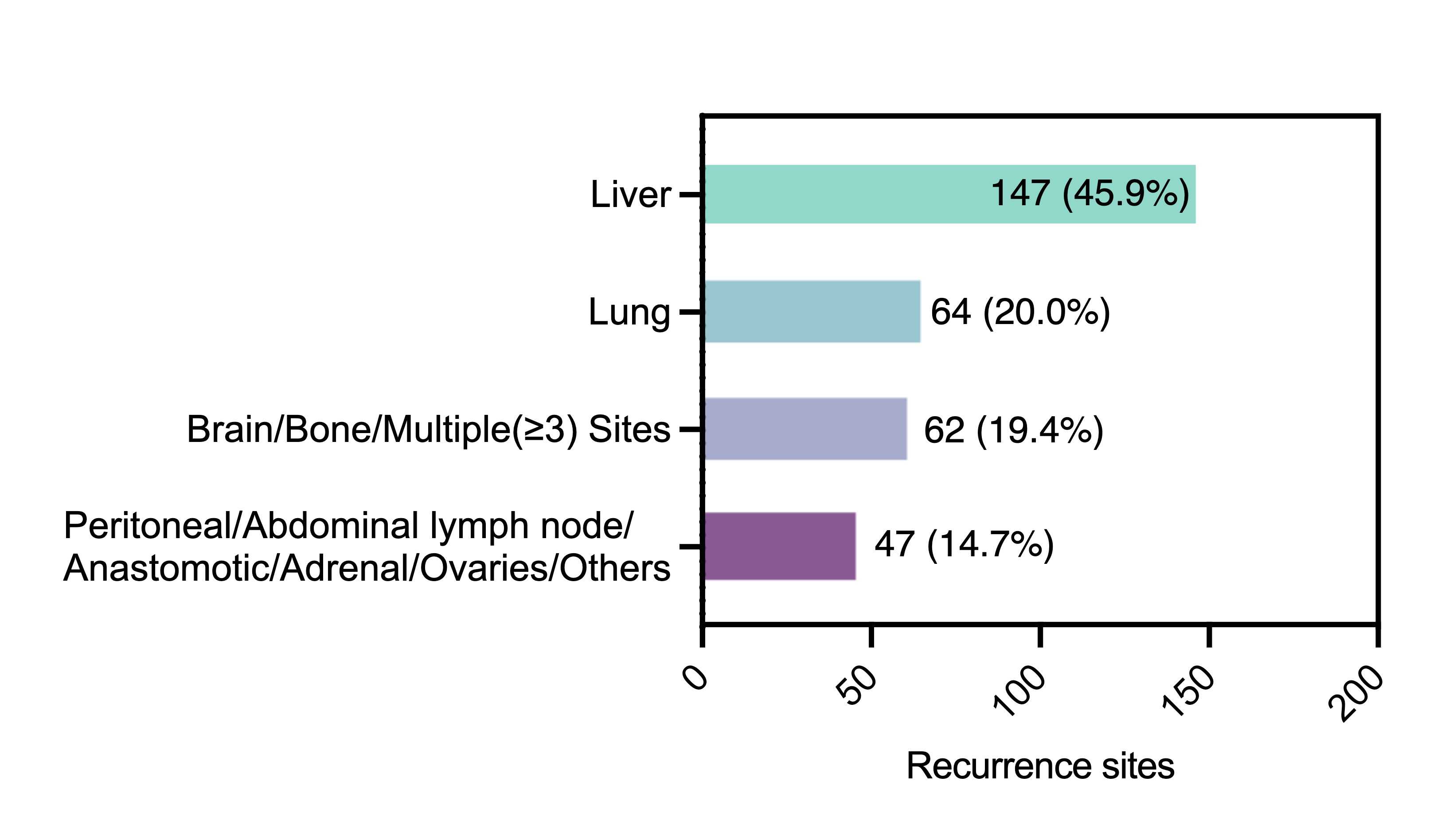

Supplement: Supplementary file 2 — Figure S2. [file CAM4-12-9559-s002.tiff]
