# Supplementary material for: Five‐year survival post hepatectomy for colorectal liver metastases in a real‐world Chinese cohort: Recurrence patterns and prediction for potential cure
Source: Cancer Med. 2023 Feb 27;12(8):9559–69. doi: 10.1002/cam4.5732 (PMC10166917; doi:10.1002/cam4.5732)
Supplement: Supplementary file 3 — Table S1. [file CAM4-12-9559-s001.docx]

**supplementary Table 1** Characteristics of patients with early and late recurrence

| Variable | | Early recurrence | Late recurrence | P-Value |
| --- | --- | --- | --- | --- |
| age (mean, year) | | 56.89±11.11 | 56.44±9.92 | 0.713 |
| gender | |  |  | **0.022** |
| male | | 65 (53.3%) | 131 (66.2%) |  |
| female | | 57 (46.7%) | 67 (33.8%) |  |
| primary CRC T stage | |  |  | 0.290 |
| t1-2 | | 12 (9.8%) | 13 (6.6%) |  |
| t3-4 | | 110 (90.2%) | 185 (93.4%) |  |
| primary CRC N stage | |  |  | **0.042** |
| n negative | | 35 (28.7%) | 79 (39.9%) |  |
| n positive | | 87 (71.3%) | 119 (60.1%) |  |
| primary CRC location | |  |  | 0.327 |
| right side | | 27 (22.1%) | 35 (17.7%) |  |
| left side | | 95 (77.9%) | 163 (82.3%) |  |
| timing of liver metastasis | |  |  | **0.005** |
| synchronous | | 83 (68.0%) | 103 (52.0%) |  |
| metachronous | | 39 (32.0%) | 95 (48.0%) |  |
| liver metastasis size (mm) |  | |  | 0.687 |
| < 50 | | 99 (81.1%) | 157 (79.3%) |  |
| ≥ 50 | | 23 (18.9%) | 41 (20.7%) |  |
| liver metastasis number | |  |  | **0.007** |
| ≤3 | | 71 (58.2%) | 144 (72.7%) |  |
| >3 | | 51 (41.8%) | 54 (27.3%) |  |
| distribution of liver metastases | | |  | **0.002** |
| unilobar | | 53 (43.4%) | 122 (61.6%) |  |
| bilobar | | 69 (56.6%) | 76 (38.4%) |  |
| RAS status | |  |  | 0.647 |
| wild-type | | 71 (60.7%) | 119 (63.3%) |  |
| mutation | | 46 (39.3%) | 69 (36.7%) |  |
| preoperative CEA (ng/ml) | |  |  | 0.138 |
| CEA <10 | | 58 (47.5%) | 111 (56.1%) |  |
| CEA ≥10 | | 64 (52.5%) | 87 (43.9%) |  |
| preoperative CA19- 9 (iu/ml) | |  |  | 0.211 |
| CA19- 9 <50 | | 84 (68.9%) | 149 (75.3%) |  |
| CA19- 9 ≥50 | | 38 (31.1%) | 49 (24.7%) |  |
| CRS | |  |  | **＜0.001** |
| low risk (<3) | | 46 (37.7%) | 120 (60.6%) |  |
| high risk (≥3) | | 76 (62.3%) | 78 (39.4%) |  |
| preoperative chemotherapy |  | |  | **0.029** |
| yes | | 88 (72.1%) | 119 (60.1%) |  |
| no | | 34 (27.9%) | 79 (39.9%) |  |
| postoperative chemotherapy | |  |  | **＜0.001** |
| no | | 45 (44.1%) | 40 (23.1%) |  |
| yes | | 57 (55.9%) | 133 (76.9%) |  |

Abbreviations: CRC, colorectal cancer; CEA, carcinoembryonic antigen; CA19-9, carbohydrate antigen 19-9; CRS, clinical risk scores
